# Modeling the impact of health care worker masking to reduce nosocomial SARS-CoV-2 transmission under varying adherence, prevalence, and transmission settings.

**DOI:** 10.1017/ice.2025.78

**Published:** 2025-08

**Authors:** Timothy D Whiteley, James Stimson, Colin S Brown, Julie V Robotham, Stephanie Evans

**Affiliations:** 1 AMR & HCAI Division, UK Health Security Agency, London, UK; 2 NIHR Health Protection Research Unit in Healthcare Associated Infections and Antimicrobial Resistance, Department of Infectious Disease, Imperial College London, London, UK; 3 NIHR Health Protection Research Unit in Healthcare Associated Infections and Antimicrobial Resistance at University of Oxford, Oxford, UK

## Abstract

**Objectives:**

To understand the scenarios where health care worker (HCW) masking is most impactful for preventing nosocomial transmission.

**Methods:**

A mathematical agent-based model of nosocomial spread with masking interventions. Masking adherence, community prevalence, disease transmissibility, masking effectiveness, and proportion of breakroom (unmasked) interactions were varied. The main outcome measure is the total number of nosocomial infections in patients and HCW populations over a simulated three-month period.

**Results:**

HCW masking around patients and universal HCW masking reduces median patient nosocomial infections by 15% and 18%, respectively. HCW-HCW interactions are the dominant source of HCW infections and universal HCW masking reduces HCW nosocomial infections by 55%. Increasing adherence shows a roughly linear reduction in infections. Even in scenarios where a high proportion of interactions are unmasked “breakroom” interactions, masking is still an effective tool assuming adherence is high outside of these areas. The optimal scenarios where masking is most impactful are those where community prevalence is at a medium level (around 2%) and transmissibility is high.

**Conclusions:**

Masking by HCWs is an effective way to reduce nosocomial transmission at all levels of mask effectiveness and adherence. Increases in adherence to a masking policy can provide a small but important impact. Universal HCW masking policies are most impactful should policymakers wish to target HCW infections. The more transmissible a variant in circulation is, the more impactful HCW masking is for reducing infections. Policymakers should consider implementing masking at the point when community prevalence is optimum for maximum impact.

## Introduction

Respiratory viruses including Severe Acute Respiratory Syndrome Coronavirus 2 (SARS-CoV-2), influenza, and Respiratory Syncytial Virus place severe pressure on healthcare systems by increasing the number of patient admissions, infections within the healthcare setting, and driving infection-related health care worker (HCW) absences.^1^ Furthermore, reportedly frequent presenteeism with respiratory illness poses a risk for nosocomial transmission within a hospital.^2^ Throughout the COVID-19 pandemic evidence of nosocomial infection was demonstrated both within and between patient and HCW populations.^3^ Patients who contract a nosocomial infection commonly experience adverse health outcomes, increased length of stay,^4^ and a greater cost due to a greater level of care required.^5^ HCW infections also contribute to the cost burden by requiring time off to isolate and recover, as well as the health impacts on the HCWs themselves.^5^ Infection prevention and control (IPC) is vitally important to protect both vulnerable patients and HCWS, and masking of HCWs both around patients and universally was a key IPC strategy throughout the COVID-19 pandemic.^6^ However, their potential impact warrants further investigation, particularly when adherence and correct usage are considered,^7,8^ and factors such as personal discomfort, loss of social cues (facial expressions and lip reading), and emotional fatigue mean that mask-wearing might not always be appropriate.^9^ Further, there are also times when HCWs cannot wear masks such as when drinking or eating lunch, and the impact of these unmasked interactions on HCW-to-HCW transmission is unclear.^10,11^ Questions remain around the scenarios where masking is most impactful and when they should be worn.^12^ Policies and behaviors may vary across hospitals with no clear consensus around the conditions in which masks should be worn.^13–15^

Mathematical models of masking in hospitals have shown both surgical and N95 masks to be an effective and cost-effective intervention method.^16,17^ In this paper we develop this theory to understand the scenarios in which masking may be the most impactful. We present an agent-based model (ABM) of the spread of a respiratory disease around a hospital containing patients and HCWs. This model is based on a hypothetical winter scenario under current patient management and HCW isolation guidelines. It is parameterized for SARS-CoV-2, although its principles and results are adaptable for influenza and other respiratory infections. We use this model to simulate the impact of alternative masking interventions on the number of hospital-acquired infections in patient and HCW populations and explore the importance of epidemiological factors such as prevalence and transmissibility as well as behavioral factors including adherence on overall reductions.

## Methods

Mathematical and computational modeling is a useful tool to understand the spread of disease within a population, particularly in scenarios where conducting real-world experiments may be impractical or unethical.^18^ Agent-based modeling considers the actions of an individual in a virtual world as they interact with and are impacted by the world around them and other agents. This paradigm enables easy implementation of complex assumptions at multiple scales that focus on the individual’s impact on the global disease burden.^18^ In the context of masking, we are considering assumptions around the individual’s actions, the hospital network, and the global disease burden. Therefore, agent-based modeling seems the optimum paradigm for this work.

We have adapted an ABM of within-hospital disease transmission and control of SARS-CoV-2 from Evans et al^6,16^ and Pople et al.^19^ The model implements three main parts: patient and health care movement; contact between agents and disease spread; and interventions. Full details are presented in appendix A but we present an overview here.

The modeled hospital is based on a typical English hospital over a hypothetical 90-day winter period. The hospital has 42 wards each with 4 bays and 6 beds per bay (1008 beds total). We assume there are 8000 HCWs, working a 12-hour alternating on/ off shift pattern, 30% of whom are ward based, representative of an NHS England (NHSE) trust.

Interactions are random based on a Poisson process with a rate accounting for the frequency and strength of the contact type. This creates a network of possible interactions which are realized each timestep. There are several possible contact types:

- Patients sharing a bay.
- Patients sharing a ward.
- HCWs visiting patients, divided into ward-based and non ward-based.
- HCWs interacting with other HCWs.

At each timestep there is a probability of a ‘contact event’ where disease may spread. These contact patterns vary for each iteration with values adapted from Pople et al^19^ (Appendix A, Table A3).

If there is a contact event, there are several factors that affect the chance of disease spread:

- The disease-specific probability weighting ( 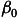 ), or the transmissibility of the disease.
- The two individuals’ probability weightings from previous infections or vaccinations (Appendix A).
- Any interventions that are in place, in this case from masking. The default values effectiveness for masking from an infected wearer is 60% and the protection for the wearer is 45%^2^ although we vary these (from 0%–100%) in the simulations shown (Table A4).

The disease course follows a S-E-I-R trajectory with the timings for each step from Bayes et al.^20^ An infected agent has a 70% chance of symptom onset.^21^ Symptomatic HCWs will isolate for three full days, aligning with the current guidance to stay off work until symptoms pass.^22^ If a patient is symptomatic, we do not assume a change in behavior such as isolation, cohort bays, or reduction of contact (see limitations). Disease ingress into the hospital occurs in two ways which both depend upon the community prevalence. Patients can be admitted into the hospital infected and HCWs can be infected in the community when they are off shift. For simplicity, we do not consider ingress or spread from family and friends visiting. Evans et al^6^ found that restricting visitors had minimal impact on overall cases.

We consider two alternative HCW masking interventions: (1) masking around patients only, and (2) universal HCW masking where HCWs mask around other HCWs as well as around patients. In no scenario are patients wearing masks as tolerance of masks by patients with respiratory symptoms is likely to be low. In all masked interactions, the HCW will wear a mask with a probability given by the adherence. In some scenarios, we vary compliance also which we define as the probability that someone will ever wear a mask. In the universal HCW masking scenario, we make one caveat that a proportion of interactions are ‘breakroom’ interactions. In these places, we know that staff may well be eating or drinking together and therefore neither staff member wears a mask. We assume 25% of interactions are unmasked ‘breakroom’ interactions at baseline, but we vary in scenario analyses to estimate its impact.

Further parametrization for the model came from Pople et al^19^ and Evans et al^6,16^ and is detailed in Appendix A. For each scenario, we run forty iterations, each with varying parameters for the various contact patterns taken from Pople et al^19^ which can be seen in Table A3 in Appendix A. Parameter summaries can be seen in Table A4.

## Results

### Overall masking impact

**Figure 1.**
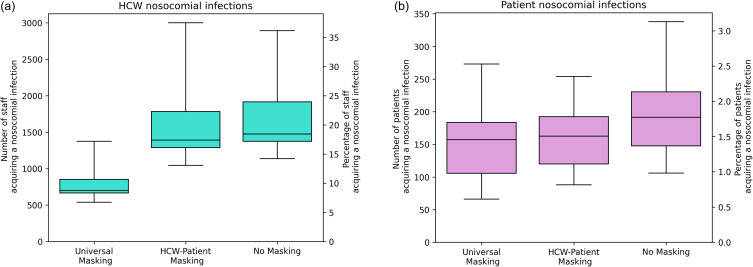
The impact of the two mask-wearing strategies on health care worker infections (a, turquoise) and patient infections (b, purple). Figure uses the default values shown in the main text and Appendix A and over the 40 iterations of different contact patterns strengths in Appendix A.

Universal masking (HCWs masking around patients and other HCWs) results in a large reduction in HCW infections but small further reduction in patient infections (Figure 1). Masking by HCWs around patients only has a small impact on the number of nosocomial HCW infections but a greater impact on nosocomial patient infections.

The percentage of HCWs nosocomially infected over the simulation period under the no masking scenario is 18.4%. Under HCW-patient masking this is reduced to 17.4% of HCW, and for universal HCW masking to 8.4% of HCWs. This corresponds to a 5.6% and 55% decrease in the number of nosocomial infections in the two masking scenarios compared to the no-masking scenario (Figure 1A). For patients, the reduction in nosocomial infections are 15% and 18% for the HCW-patient and universal masking scenarios compared to no masking (Figure 1B).

The median onward infections from each infected HCW to other HCWs is 0.26, 0.43, and 0.45 for universal masking, HCW-patient masking and no masking, tying in with Evans et al.^6^ The average number of patient infections per HCW infected is 0.011, 0.011, and 0.021 for the three scenarios (Appendix B, Figure B1).

### Transmissibility and prevalence

**Figure 2.**
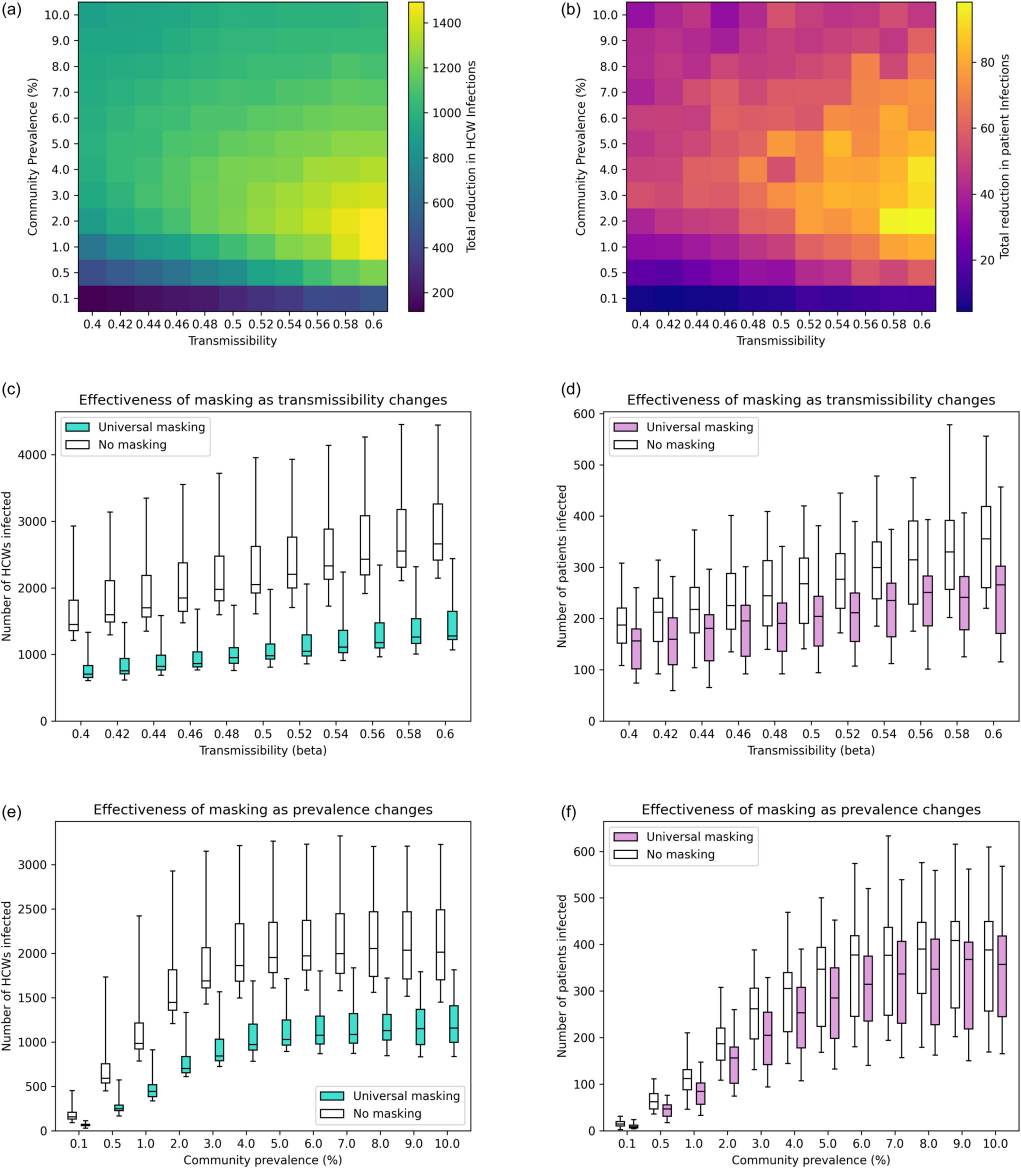
The impact of changing disease transmissibility and community prevalence on infections and masking impact. (a,b) The reduction in mean number of infections for health care workers (HCWs) and patients. (c, d) Boxplots of the distribution of estimates of HCW and patient infections with and without masking as transmissibility increases. The community prevalence is at the default value of 2%. (e, f) Boxplots of the distribution of estimates of HCW and patient infections with and without masking as prevalence increases. The transmissibility is the default value of 0.4. For all subfigures, all other parameters use the default values shown in the main text and Appendix A and over the 40 iterations of different contact patterns strengths in Appendix A.

When disease transmissibility and community prevalence are varied, we observed that masking was most impactful when the transmissibility is highest ( 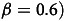 but community prevalence moderate (1-2% for HCWs, 2% for patients, Figure 2a,b).

As the transmissibility of the virus increases (Figure 2c,d), universal HCW masking always leads to lower infection numbers compared to no masking, but the impact on patient infections is more modest. When 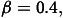 masking prevents 746 out of 1447 HCW infections (a 52% reduction). When transmissibility increases to 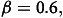 masking prevents 1383 out of 2662 HCW infections (also a 52% reduction). The proportion of infections prevented by masking stays constant, but the total number of infections are higher and therefore masking is more impactful. For patients, the number of infections prevented goes from 31 out of 187 (17% reduction) when 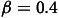 to 90 out of 356 (25% reduction) when 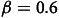 . Masking is also shown to be impactful across different infectivity periods (Appendix C).

As community prevalence increases, the total numbers of hospital-acquired infections increases and then plateaus (Figure 2e,f). HCW infections plateau at a lower community prevalence than for patients. The percentage of patient infections prevented by masking is 43% when prevalence is 0.1%, 25% when prevalence is 1%, and 8% when prevalence is 10%. For HCWs, it is 59%, 55%, and 43%, respectively.

## Adherence

**Figure 3.**
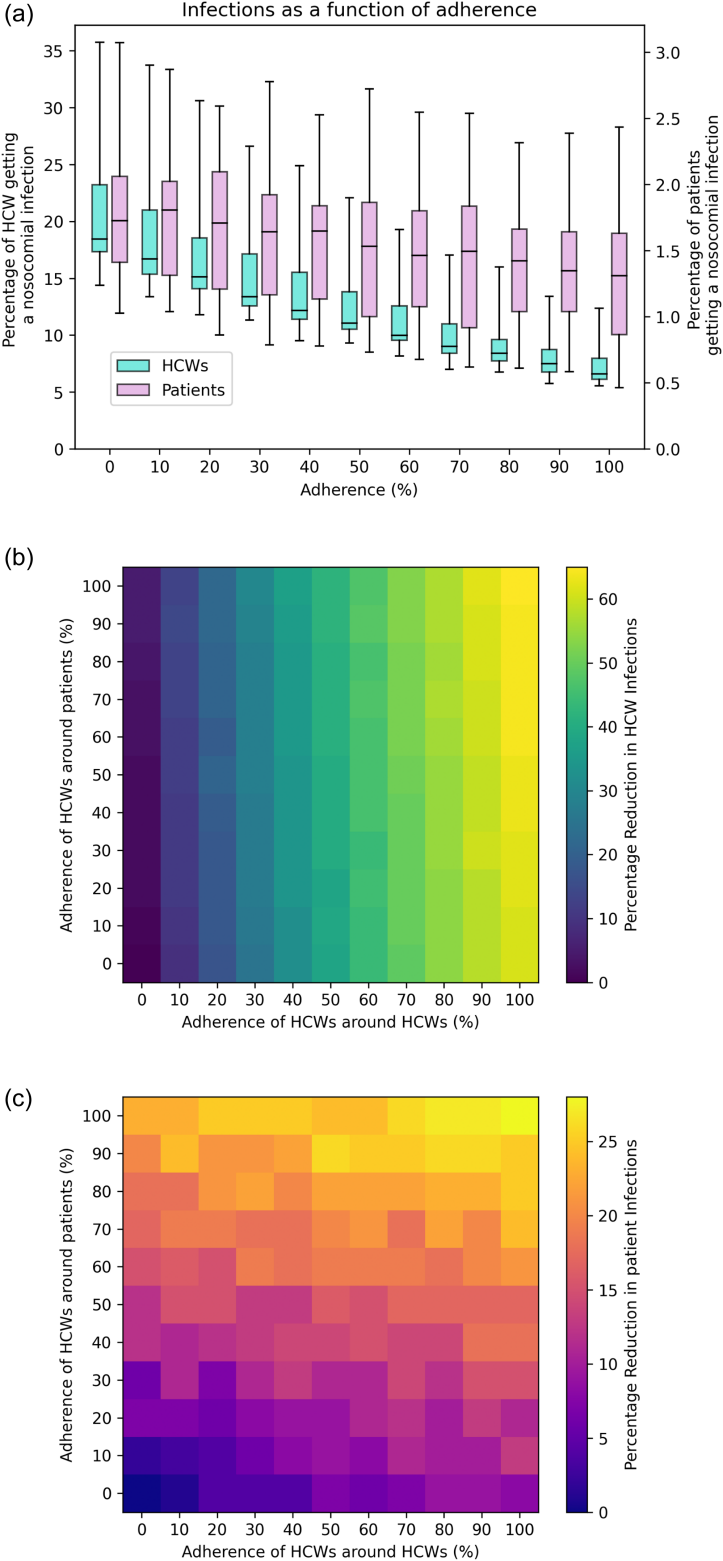
(a) Hospital-acquired infections for health care workers (HCWs) (turquoise/ left) and patients (purple/ right). Adherence to universal masking is the probability a mask is worn, incorporating both HCW-HCW interactions and HCW-patient interactions. (b) and (c) Heatmaps showing how this adherence varies by type of adherence for HCW infections (b) and patient infections (c). All other parameters use the default values shown in the main text and Appendix A and over the 40 iterations of different contact patterns strengths in Appendix A.

Increasing adherence reduces the overall burden of infections (Figure 3a). As adherence increases, HCW infections decrease more substantially than patient infections. Adherence around the person you are trying to protect is most impactful overall, ie adherence around patients has the strongest impact on patient infections (Figure 3b), and adherence around HCWs has the strongest impact on HCW infections (Figure 3c).

The decrease in overall infections is roughly linear with respect to masking adherence (Figure 3a). At 100% adherence the median percentage of HCWs infected over the three months is 6.6%, whereas with 0% adherence, it is 18.5%. This is a decrease of 11.9% of HCWs (949 HCWs). At this rate, each percentage of adherence to universal masking prevents 9.5 hospital-acquired HCW infections. For patients, the median decrease is 1.7% of patients to 1.3% of patients which is a reduction in 43 patient infections.

There is no difference in the model between compliance (the probability that someone is a masker) and adherence (the probability a masker masks) (Appendix B).

### Breakroom interactions

**Figure 4.**
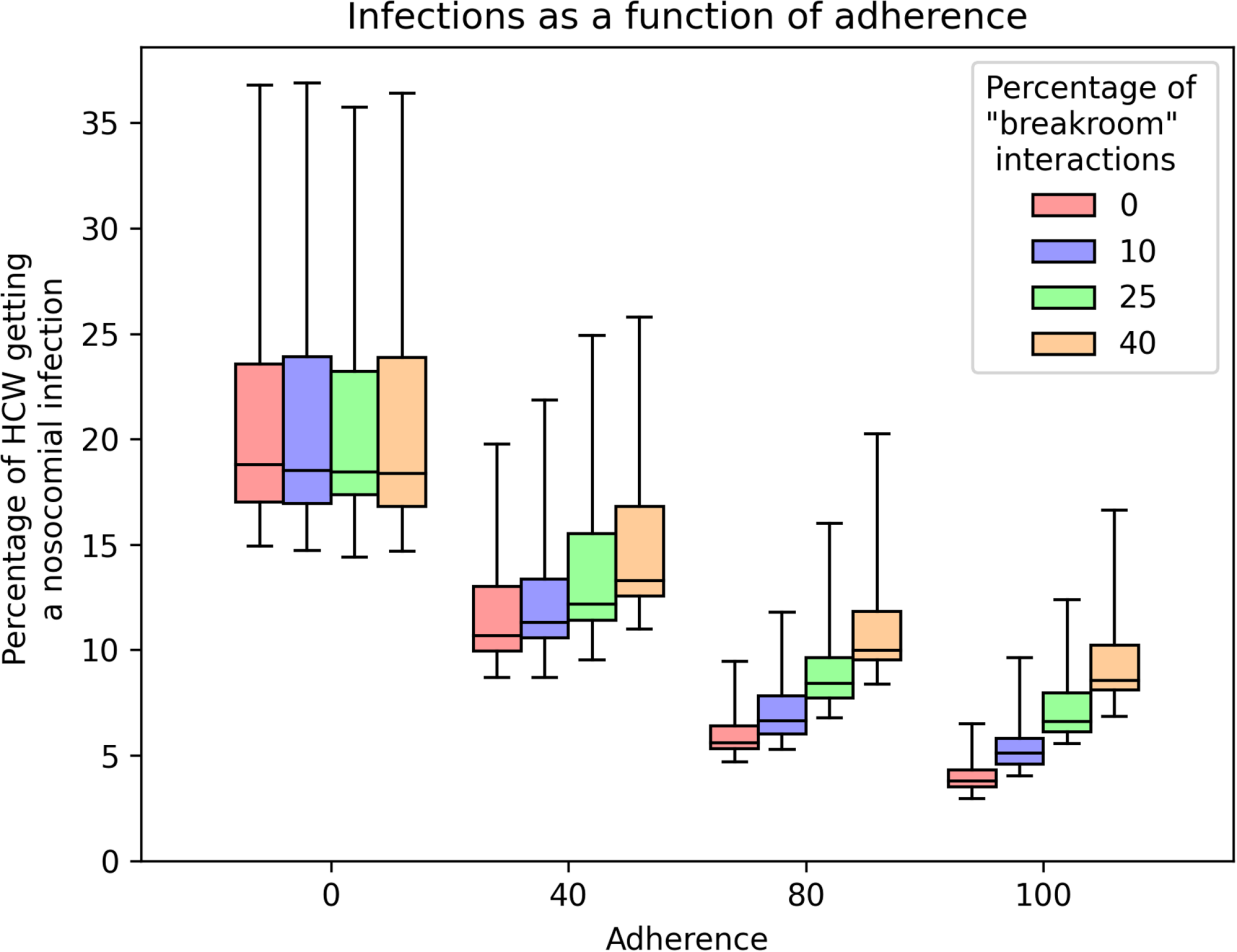
Comparison of health care worker nosocomial infections as adherence (x-axis) and proportion of time in breakroom changes (color and increasing L-R). All other parameters use the default values shown in the main text and Appendix A and over the 40 iterations of different contact patterns strengths in Appendix A.

Even where the proportion of breakroom interactions is high (40% of interactions unmasked), increased adherence outside of a breakroom remains an effective way to reduce nosocomial transmission within the HCW population (Figure 4). With perfect adherence (100%), the mean percentages of HCWs that are infected in hospital are 4%, 5.3%, 7.3%, and 9.4% for 0%, 10%, 25% and 40% unmasked ‘breakroom’ interactions. By comparison, if the adherence is 40% then the percentages of HCWs infected are 11.8%, 12.5%, 13.7%, and 15.0%, respectively. The baseline unmasked scenario (0% adherence) gives on average a 20.7% infection rate.

### Mask effectiveness

**Figure 5.**
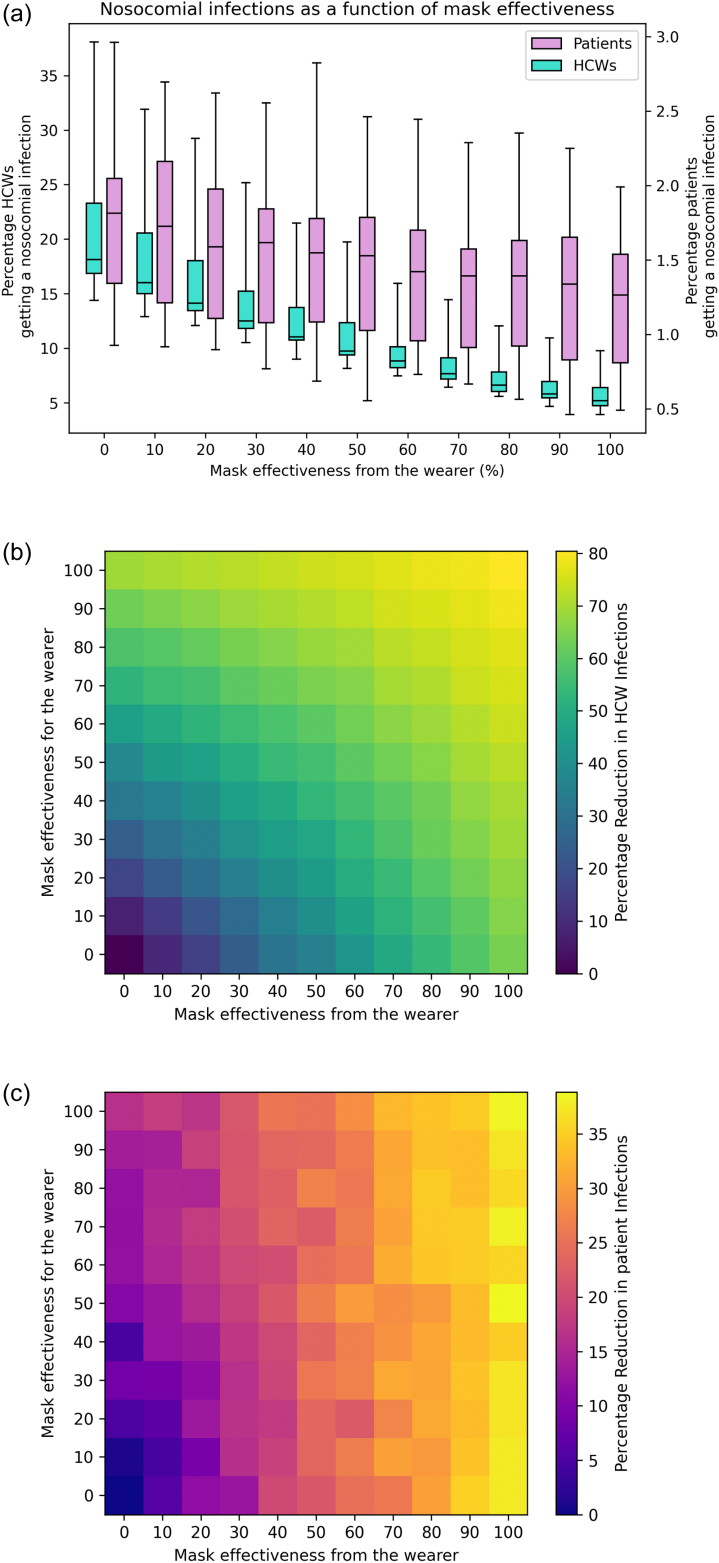
(a) Percentage of health care workers (turquoise/left) and patients (pink/right) getting a hospital-acquired infection as effectiveness from the wearer changes. In this figure, we assume that effectiveness for the wearer is 75% of the effectiveness from the wearer. (b) and (c) the percentage reduction in the number of infections for health care workers and patients. All other parameters use the default values shown in the main text and Appendix A and over the 40 iterations of different contact patterns strengths in Appendix A.

As mask effectiveness increases, the reduction in infections follows an approximately linear trend (Figure 5a). Setting mask effectiveness from the wearer to 50%, 60% (default), 70%, and 80% reduces the median percentage of HCWs that are nosocomially infected from 18.1% (no masking) to 9.7%, 8.8%, 7.6%, and 6.6%, respectively.

Protection from the wearer is most important if patient infections are considered, whereas protection for the wearer (the HCW) will have minimal onward impact on patient infections (Figure 5b,c). For HCW infections, protection for and from the wearer provide very similar impact.

## Discussion

In this paper we have shown that masking, where appropriate, can significantly reduce nosocomial infections, protecting patients and HCWs, in line with previous modeling studies.^6,16^ We have also shown that increasing effectiveness, compliance, and adherence all have a small but important impact on the overall burden of disease in the hospital and that there are optimum regions of prevalence and transmissibility where masking is most effective as a preventative measure. We also demonstrated that when community prevalence is high, masking effectiveness reduces. This is not necessarily because there will be less disease in the hospital but because HCWs are increasingly likely to contract the infection from the community rather than from the hospital. Conversely, as transmissibility increases, the internal chains of transmission will be longer and therefore masking is more effective at stopping such a chain as early as possible. Together, this could imply that masking may be especially effective in the earlier stages of an epidemic where community prevalence is lower but the disease can quickly spread through a susceptible population as demonstrated during the COVID-19 pandemic.^3^

The model parameterization shows that the chains of transmission within the hospital are often short. Lindsey et al^3^ estimate a median of three links in waves 1 and 2. In our model, this is even shorter due to vaccination and previous infection-reducing transmissibility. Short chains of transmission mean there are not significant non-linearities, ie ‘tipping points’. Rather, we see that every increase in compliance rates leads to benefits in terms of preventing nosocomial spread. Moreover, we found that masking is directly impactful, rather than preventing risks of ongoing transmission. HCW masking around patients does little to protect HCWs and universal HCW masking has a small knock-on effect on overall patient infection. Similarly, the protection masking brings for the wearer does not prevent patient infections as they are unmasked. For HCWs, the HCW-to-HCW transmission route is the dominant source of nosocomial HCW infections– therefore a masking policy that aims to tackle staff sickness and absences should target this route of transmission rather than focusing on protecting HCWs from patient infections.

We have presented a range of values for masking effectiveness as there is still uncertainty in the literature around these values, particularly in real-world settings where incorrect and intermittent usage may impact the perceived effectiveness of the mask.^23–28^ We have shown that mask effectiveness and adherence have, in the model, a very similar impact on the reduction of infections. Transmission is prevented by having a mask in place and that the mask is effective at blocking infection. Therefore, the overall effectiveness of masking to prevent infections can be roughly understood as the product of effectiveness and adherence. We have demonstrated that increasing adherence has an impact on overall nosocomial infections, however, there are other important factors that affect masking’s overall value, such as social interaction, wearer comfort and financial cost.^9,22^ This paper presents only one side of the wider discussion.

## Limitations

The results presented are from a hypothetical modeling study, based on COVID-19. This has allowed us to present general trends and elicit overarching relationships between the scenarios considered and the impact of masking.

A limitation of ABMs compared to other modeling approaches is the requirement of detailed assumptions resulting in additional parametrization, computational demand, and difficulty in analytical interpretation. We have, however, made parsimonious assumptions and used Monte-Carlo simulation to present our analysis.

Although the model parameters were previously calibrated to data from NHS England and the SARS-CoV-2 Immunity and Reinfection EvaluatioN study,^6,19^ there is high uncertainty around parameters such as the number of interactions that individuals make during a day, in particular HCW-HCW interactions. If the real-world value of this parameter is higher (lower) than assumed, then the number of infections due to this route will be correspondingly higher (lower) and the importance of HCW-HCW masking will be greater (less). We present results from a range of parameter sets where transmission rates within and between populations of HCWs and patients produce estimates within the bounds of observed data^6^ and, in all the parameter sets considered, the overall trajectories from masking are retained.

Differential masking strategies of HCWs around known cases is not considered under this model and it is assumed that there are no changes in behavior around known COVID-19 cases. If included, this might change the impact of masking with better adherence improving impact and non-masking around undetected cases lowering the impact. However, in our study, the impact of masking around patients on HCW infections appears to be minimal as the greatest transmission risk is from other HCWs. This is supported by genomic analyses.^3^

We have not modeled different mask types, only different strategies of masking under a range of estimates of effectiveness, with the baseline parameterization being representative of fluid-resistant surgical masks. With further effectiveness evidence, the expected impact of other mask types on nosocomial infections could be estimated from these results. However, the impact of other mask types (eg FFP3) is not limited to just effectiveness of masking but also impacts on compliance and adherence that are not well captured in the literature.

Building on this paper, future research could explore additional factors that influence the impact of masking. For example, finding the optimum mask-wearing period during the course of an epidemic, and if the results presented in this paper are robust in situations where prevalence or policy may fluctuate over time. Alternatively, if more detail around hospital structure and ward types were available, models could determine whether there are certain wards or scenarios where masking may be more impactful.

In conclusion, this study uses a computational model to explore factors that affect the impact of HCW masking on nosocomial infections. We found that masking reduces the number of nosocomial infections across all effectiveness and adherence ranges considered and that every effort made to increase adherence can reduce the number of nosocomial infections. When considering the introduction of a masking policy, care should be taken to optimize the timing for maximum impact using real-time data and feedback loops to inform mask policy decisions.

## Supporting information

Whiteley et al. supplementary material 1 Whiteley et al. supplementary material

Whiteley et al. supplementary material 2 Whiteley et al. supplementary material

Whiteley et al. supplementary material 3 Whiteley et al. supplementary material

## Supplementary material

The supplementary material for this article can be found at https://doi.org/10.1017/ice.2025.78
