# Supplementary material for: Modeling the impact of health care worker masking to reduce nosocomial SARS-CoV-2 transmission under varying adherence, prevalence, and transmission settings
Source: Infect Control Hosp Epidemiol. 2025 Jun 27;46(8):812–8. doi: 10.1017/ice.2025.78 (PMC12483623; doi:10.1017/ice.2025.78)
Supplement: Whiteley et al. supplementary material 1 — Whiteley et al. supplementary material [file S0899823X25000789sup001.docx]

**Appendix B: Extra figures:**

(a)
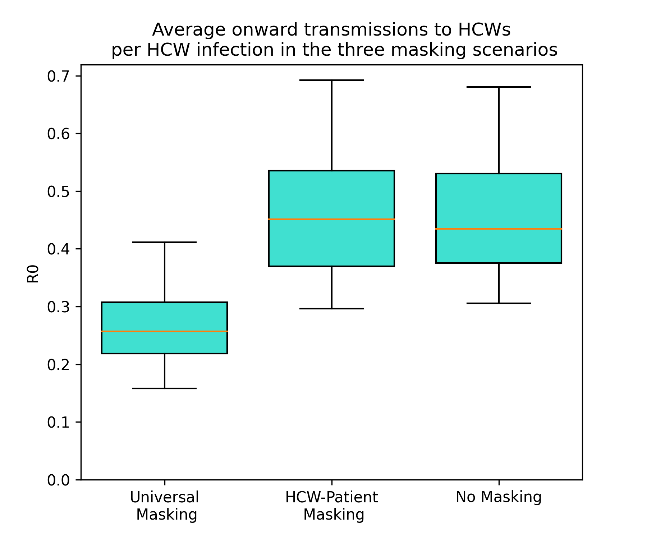
(b)
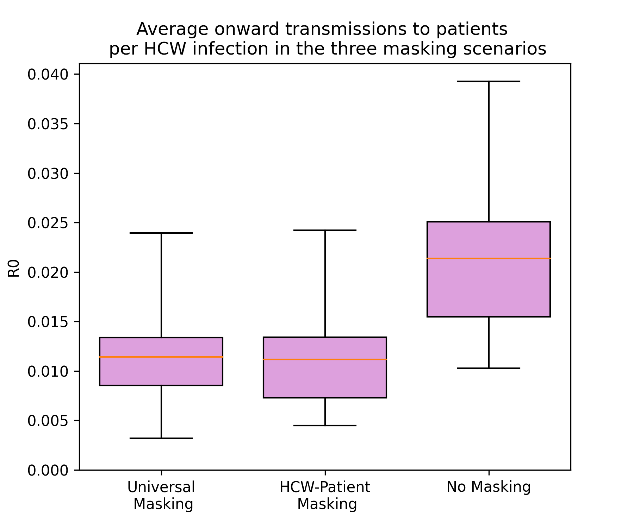


*Figure B1: Boxplots of the average number of onward infections per infected health care worker under the three masking scenarios broken into HCW infections (a) and patient infections (b). All parameters use the default values shown in the main text and appendix A and over the 40 iterations of different contact patterns strengths in appendix A.*


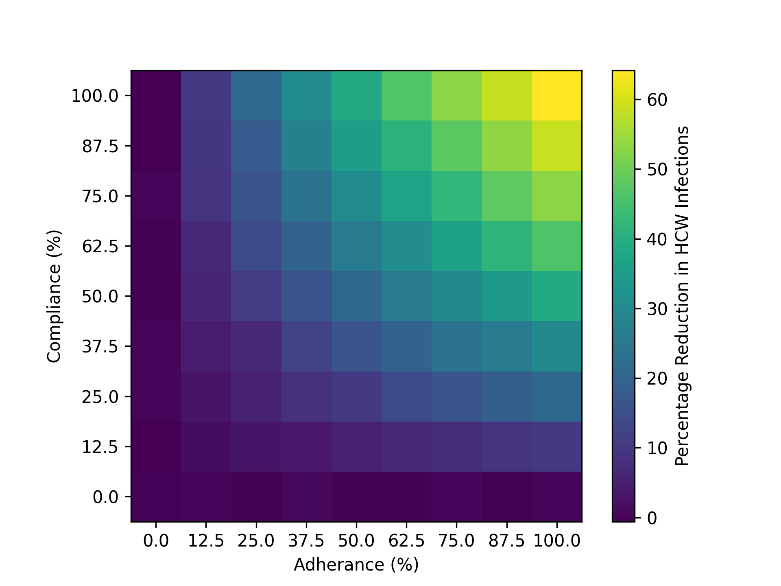


Figure B2: Plot of adherence versus compliance and the percentage reduction in the number of health care worker infections. All other parameters use the default values shown in the main text and appendix A and over the 40 iterations of different contact patterns strengths in appendix A.
