# Supplementary material for: Modeling the impact of health care worker masking to reduce nosocomial SARS-CoV-2 transmission under varying adherence, prevalence, and transmission settings
Source: Infect Control Hosp Epidemiol. 2025 Jun 27;46(8):812–8. doi: 10.1017/ice.2025.78 (PMC12483623; doi:10.1017/ice.2025.78)
Supplement: Whiteley et al. supplementary material 2 — Whiteley et al. supplementary material [file S0899823X25000789sup002.docx]

**Appendix C: Varying the infectivity period.**

In this appendix we demonstrate that universal masking is reduces transmission across a range of infectious periods of the disease (the time in days from I to R).

In the model we generalise the infectious period as $x+\mathrm{Gamma}\left( 2,2.1 \right)$ where the default value of $x=2$gives a mean time to recovery of 6.2 days. We vary $x$ from 0 to 4 to give a mean infectivity period of between 4.2 and 8.2 days.

In all scenarios, masking is can reduce the number of nosocomial infections (Figure C1), especially among HCWs, and the impact increases with the length of the infectious period. For HCWs, masking prevents 52% and 56% infections for the lowest and highest infectious period respectively.

The impact of masking to protect patients also increases as the infectious period increases. The infections prevented goes from 10% of patient infections prevented when universal masking is in place when the mean is 4.2 days to 28% when the mean is 8.2 days.

| 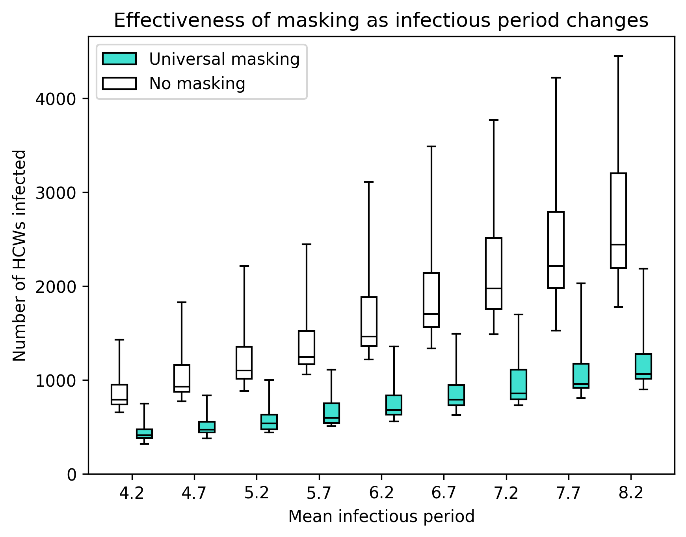 | 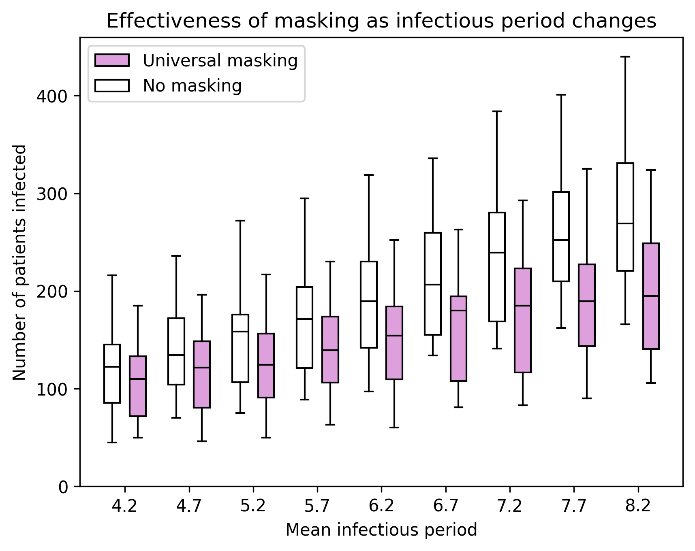 |
| --- | --- |
| *Figure C1: Boxplots of numbers of nosocomial infections with and without universal masking as the mean infectious period varies for HCWs (turquoise, left) and patients (pink, right).* | |
