# Supplementary material for: Modeling the impact of health care worker masking to reduce nosocomial SARS-CoV-2 transmission under varying adherence, prevalence, and transmission settings
Source: Infect Control Hosp Epidemiol. 2025 Jun 27;46(8):812–8. doi: 10.1017/ice.2025.78 (PMC12483623; doi:10.1017/ice.2025.78)
Supplement: Whiteley et al. supplementary material 3 — Whiteley et al. supplementary material [file S0899823X25000789sup003.docx]

**Appendix A: Model detail and parameters**

**Overview**

In this paper we use an agent-based model of disease transmission that is based on Pople et al [1] and Evans et al [2]. In the model, health care workers (HCWs) and patients interact and the disease spreads between them. This model is implemented in python using the underlying MESA package [3].

We assume a hospital of 1008 beds [1,2] divided up into 42 wards each of which contains four bays with six beds. This is designed to fit an arbitrary medium sized UK hospital.

Time is divided into 6 ticks per day and each timestep, a number of things:

- Any isolating HCWs will return to the system.
- Each agent will move. This includes staff starting/ finishing work, and patients being discharged.
- New patients will be admitted to hospital.
- The disease will spread.

**HCWs**

We assume 8000 HCWs with non-specific assignments but an 30% chance of being ward based. HCWs work a 12 hour on/ off pattern, 7 days a week. We do not account for weekends or holidays for staff.

**Patients**

New patient cases are admitted each timestep as a Poisson process with a rate of 120 per day to fit with rough estimates from a UK hospital. They are allocated a bed randomly from any that are free.

New patient agents are created as they join the hospital. They are given parameters for age, sex and length of stay. Age is taken from a lognormal distribution with shape 5 and rate 70, as per Evans et al [2]. Sex is randomly assigned with 50% probability. Length of stay is assigned according to their age and gender as a lognormal function with parameters as in Table A1.

To estimate this length of stay, data for all hospitalisations in 2019 was taken from HES APC as an example of the last year unaffected by covid. We group records hospital episodes into spells in order to estimate total length of stay. Any spell with a LOS of 0 is excluded. Various GLM models were fit to this data including gamma, lognormal and Poisson. A lognormal was chosen as the model with the lowest AIC.

| *Table A1: Parameter values for length of stay lognormal distribution.* | |
| --- | --- |
| **Definition** | **Value** |
| Shape function | 0.887 |
| Rate | 0.272 |
| Coeffcient of age | -0.0129 |
| Coeffcient of sex (female) | 0.0512 |

**Disease**

The model is parametrised for COVID-19, but the setup is applicable to any respiratory disease, and designed to be adaptable should the model which to be modified.

The disease follows an E-I-R-S trajectory. E to I is a lognormal distribution with mean 1.6112 and standard deviation 0.47238 from Bayes et al [4]. I to R is given by $2+\Gamma(2,2.1)$ to fit with Quilty et al [5]. The R-S trajectory is given by N(100,10) as this fits with [6-8].

When someone returns to susceptible, they are still protected somewhat by previous infections, as they are from vaccination. Protection is assumed to be 10%. This protection is the reduction in the probability of infection.

$$P\left( will be infected \right)=p\left( would be infected \right)\cdot(1-\mathrm{protection})$$

The probability of symptom onset is 70%. The base transmissibility of the disease could be absorbed into other parameters but we keep it as a separate attack rate. The default is $\beta=0.4$ as in the community model of Evans et al [2]. This is varied in simulations.

Practically, when someone is infected this creates a new ‘infection’ agent which is attached to that person agent. Each timestep, the infection will evaluate whether to change state given the time or whether the person will ‘recover’. Using this formulation allows us to easily switch disease dynamics for future uses without having to adapt any of the underlying architecture of the model.

**Contact**

Contact is based on the idea of a Poisson process where contacts occur at a certain rate between individuals and each contact has the probability of spreading the disease. If there is a rate of contact $\lambda$ and probability of transmission $p$then the probability of disease transmission in a timestep of length $t$is given by $1-\lambda\cdot p\cdot t$. We can therefore consider $\beta=\lambda\cdot p$ as a hyperparameter for each scenario.

This approach has a few advantages. It is designed to be computationally fast as we do not track the specifics of individual movements or visits. Using this formulation also means that we do not need a new contact pattern for each timestep (although we could do if we wanted to) yet still allowing for random interactions. It is straightforward to amend the rate of contact or the probability of disease transmission for the specific disease/ contact type/ situation. Lastly, a Poisson process is additive, so people contact each other via multiple contact events then we can sum the rates.

To set this up, each agent is assigned a number of different agents with whom they interact regularly. For each interaction there is a $\beta$ superparameter estimating both the rate that they will make contact one another and the base probability of transmission given contact.

There are a number of different types of contact that we account for. For each type of interaction we assign the number of contacts that each agent will have and the beta rate for each contact. Contacts are built from the following assumptions:

- HCWs visiting patients.
- Patients sharing a bay/ ward.
- HCWs interacting with other HCWs.

We do not assume any contact via visiting family members.

Estimates for the number and rate at which contacts occur comes from Pople et al [1]. We ensure that the product of the number of contacts and the rate remains constant, while slightly adjusting the parameters for speed optimization, maintaining the inverse proportionality. The number of contacts that we consider for each type of contact are as below:

| *Table A2: Number of contacts assigned in the model according to type.* | |
| --- | --- |
| Patient to patient ward contacts | 13 |
| Number of HCWs allocated per patient (non ward based) | 30 |
| Number of HCWs allocated per patient (ward based) | 18 |
| HCW to HCW ward contacts | 18 |

For the estimates for the rate at which contacts occur, we consider a range of 40 parameter values that were used to account for the uncertainty in these key parameters. The values used for the can be seen in Table 3.

If the Poisson process generates a contact event then each contact event then has a disease specific probability of transmission $(\beta_{0}$). For each person, if they would have multiple infections in a timestep, we randomly chose one.

**Community infections**

In winter 2023-2024 the prevalence of COVID-19 in the community went from 1.5% to 4.2% [9-11]. We therefore consider a default prevalence of 2% but explore a range of fixed values around this.

The probability that a new hospital case will have covid-19 will be proportional to the community prevalence. Evans et al [8] use admissions to estimate community prevalence. Working backwards from their estimates we get that the probability of a new case having covid-19 is 4.58*community prevalence. For patients who are infected on arrival, we give them an infection time as a random period somewhere in the preceding 72 hours.

HCWs may also be infected from the community. We assume they have a number of contacts during their 12 hour work period. The rate of contact that HCWs have in the community comes from Evans et al [2] who estimate the total number of contacts expected and the proportion that are expected to be in the community. The probability that their contact is infected is given by the community prevalence and the probability that the infection spreads from each interaction is $\beta_{0}=0.4$ also from Evans et al [2]. The values are listed in Table 3 below.

**Vaccination and previous infections**

We assume there have been a number of previous vaccination and infection events that have occurred in the years since the vaccine began. For simplicity we assume there are up to 6 of these, (eg 3-4 vaccinations and 1-2 infection events). We assume each occurs with a probability of 60%, and each giving a protection of 10% as in Evans et al [2] citing Andrews et al [12]. This means the number of previous events is given by $n\sim bin(6,0.6)$ and the protection is given by $protection=1-\left( 1-0.1 \right)^{n}$. We do not assume waning of the vaccination or previous infections in this scenario.

**Isolation**

We assume no regular asymptomatic testing or testing when symptom onset occurs. However, if a HCW is symptomatic then they will isolate. The current UK guidance [13] states they should stay off work until symptoms are passed so we assume a three day isolation period. In HCWs who are dealing with severely immunosuppressed patients the guidance is stricter but we are not focusing on this area in this paper.

**Masking defaults**

Where otherwise stated these are the default parameters used for mask effectiveness and usage.

- Adherence by HCWs around other HCWs: 75%
- Adherence by HCWs around patients: 75%
- Effectiveness of the mask *from* the wearer: 60% (from [2])
- Effectiveness of the mask *for* the wearer: 45% (or 75% of the effectiveness from the wearer)

| *Table A3: Contact/ transmission rates for the different contact types and the number of community contacts. The forty iterations are the range of parameter values over which the model runs.* | | | | |
| --- | --- | --- | --- | --- |
| **Patient to patient (sharing a bay)** | **Patient to patient (sharing a ward)** | **HCW to patient** | **HCW to HCW** | **Community contacts within 12hr rest period.** |
| 0.00254 | 0.00000531 | 0.000380 | 0.00434 | 0.591 |
| 0.00127 | 0.0000146 | 0.000437 | 0.00374 | 0.705 |
| 0.00148 | 0.0000181 | 0.000344 | 0.00442 | 0.675 |
| 0.00186 | 0.0000864 | 0.000158 | 0.00450 | 0.754 |
| 0.00220 | 0.0000261 | 0.000338 | 0.00502 | 0.518 |
| 0.00165 | 0.0000211 | 0.000169 | 0.00497 | 0.630 |
| 0.00207 | 0.0000896 | 0.000328 | 0.00400 | 0.895 |
| 0.00121 | 0.0000438 | 0.000276 | 0.00588 | 0.387 |
| 0.00207 | 0.00000757 | 0.000364 | 0.00395 | 1.07 |
| 0.000251 | 0.000249 | 0.000201 | 0.00483 | 1.04 |
| 0.00102 | 0.000215 | 0.000230 | 0.00477 | 0.870 |
| 0.000149 | 0.000200 | 0.000344 | 0.00442 | 0.675 |
| 0.00130 | 0.000194 | 0.000141 | 0.00888 | 0.656 |
| 0.000698 | 0.000189 | 0.000223 | 0.00704 | 0.417 |
| 0.000260 | 0.000184 | 0.000276 | 0.00588 | 0.387 |
| 0.00144 | 0.000168 | 0.000229 | 0.00718 | 0.505 |
| 0.000265 | 0.000160 | 0.000338 | 0.00502 | 0.518 |
| 0.000938 | 0.000140 | 0.000584 | 0.00620 | 0.473 |
| 0.00118 | 0.000139 | 0.000219 | 0.00596 | 0.764 |
| 0.000368 | 0.000109 | 0.000294 | 0.00845 | 0.434 |
| 0.000224 | 0.0000939 | 0.000328 | 0.00400 | 0.895 |
| 0.00127 | 0.0000146 | 0.000437 | 0.00374 | 0.705 |
| 0.00148 | 0.0000181 | 0.000344 | 0.00442 | 0.675 |
| 0.00186 | 0.0000864 | 0.000158 | 0.00450 | 0.754 |
| 0.00220 | 0.0000261 | 0.000338 | 0.00502 | 0.518 |
| 0.00165 | 0.0000211 | 0.000169 | 0.00497 | 0.630 |
| 0.00207 | 0.0000896 | 0.000328 | 0.00400 | 0.895 |
| 0.00121 | 0.0000438 | 0.000276 | 0.00588 | 0.387 |
| 0.00207 | 0.00000757 | 0.000364 | 0.00395 | 1.07 |
| 0.000251 | 0.000249 | 0.000201 | 0.00483 | 1.04 |
| 0.00102 | 0.000215 | 0.000230 | 0.00477 | 0.870 |
| 0.000149 | 0.000200 | 0.000344 | 0.00442 | 0.675 |
| 0.00130 | 0.000194 | 0.000141 | 0.00888 | 0.656 |
| 0.000698 | 0.000189 | 0.000223 | 0.00704 | 0.417 |
| 0.000260 | 0.000184 | 0.000276 | 0.00588 | 0.387 |
| 0.00144 | 0.000168 | 0.000229 | 0.00718 | 0.505 |
| 0.000265 | 0.000160 | 0.000338 | 0.00502 | 0.518 |
| 0.000938 | 0.000140 | 0.000584 | 0.00620 | 0.473 |
| 0.00118 | 0.000139 | 0.000219 | 0.00596 | 0.764 |
| 0.000368 | 0.000109 | 0.000294 | 0.00845 | 0.434 |

| *Table A4: Summary of parameters used in the model, the default value and whether it is fixed throughout, varies in scenarios, or varies in iterations.* | | |
| --- | --- | --- |
| **Parameter/ assumption** | **Fixed/ varying** | **Reference** |
| The hospital has 1008 beds, divided into 42 wards, each containing four bays with six beds. | Fixed | Pople et al [1], Evans et al [2] |
| There are 8000 HCWs | Fixed |  |
| HCWs are ward-based with 30% probability | Fixed |  |
| HCWs work in 12-hour shifts on a continuous 7-day cycle | Fixed |  |
| HCWs isolate for 3 days if symptomatic | Fixed | Estimated from current UK guidance [13] |
| Patients are randomly assigned to any free bed upon admission. | Fixed | Model simplification |
| Age distribution follows a lognormal distribution with shape 5 and rate 70. | Fixed | Pople et al [1], Evans et al [2] |
| 120 patients are admitted per day, following a Poisson distribution | Fixed | Based on Hospital Episode Statistics (HES APC) |
| Length of stay follows a lognormal distribution with shape $0.887$ and rate $0.272\times exp(-0.0129\times age+0.0512\times female)$. | Fixed | Pople et al [1], Evans et al [2] |
| The probability of a new patient being infected with COVID-19 is 4.58*community prevalence. | Fixed |  |
| Patients admitted infected are assigned a random infection onset time within the previous 72 hours. | Fixed | Simplifying assumption |
| The model follows an E-I-R-S disease trajectory. | Fixed | Model assumption |
| The E to I transition follows a lognormal distribution with a mean of 1.6112 days and a standard deviation of 0.47238. | Fixed | Bayes et al [4] |
| I to R disease transition distribution is 2 + Gamma(2,2.1) | Fixed | Quilty et al [5] |
| The R to S transition is normally distributed with mean 100, standard deviation 10. | Fixed | Assumed based on literature [6–8] |
| Probability of symptom onset is 70%. | Fixed | Shang et al [14] |
| Disease protection from prior infection is 10% | Fixed | Andrews et al [12] |
| Initial protection from previous infection and vaccination is given by $1-\left( 1-0.1 \right)^{n}$ where $n\sim bin(6,0.6)$. | Fixed | Andrews et al [12] |
| Base transmissibility ($\beta_{0}$) = 0.4 | Varies in scenarios | Adapted from Evans et al [2] |
| Number of patient-to-patient contacts (ward): 13 | Fixed | Adapted from Pople et al [1] |
| HCWs per patient (ward-based): 18 | Fixed |  |
| HCWs per patient (non-ward-based): 30 | Fixed |  |
| HCW to HCW contacts: 18 | Fixed |  |
| Community prevalence: 2% | Varies in scenarios | Derived from UKHSA 2023-2024 prevalence estimates [9–11] |
| Default mask adherence (HCWs with patients): 75% | Varies in scenarios | Pople et al [1], Evans et al [2] |
| Default mask adherence (HCWs with HCWs): 75% | Varies in scenarios |  |
| Default mask effectiveness (for wearer): 45% | Varies in scenarios |  |
| Default mask effectiveness (from wearer): 60% | Varies in scenarios |  |
| Default unmasked (breakroom) interactions: 25% | Varies in scenarios | Assumption |
| Number of community contacts are varied in Table A3.  Probability of community transmission per contact is 0.4*community prevalence. | Varies in 40 iterations | Evans et al [2] |
| Transmission rate per contact type varies as in Table A3 | Varies in 40 iterations |  |

**References**

[1] Pople D, Monk EJM, Evans S, Foulkes S, Islam J, Wellington E, et al. Burden of SARS-CoV-2 infection in healthcare workers during second wave in England and impact of vaccines: prospective multicentre cohort study (SIREN) and mathematical model. BMJ. 2022;378. doi:10.1136/bmj-2022-070379.

[2] Evans S, Stimson J, Pople D, White PJ, Wilcox MH, Robotham JV. Impact of interventions to reduce nosocomial transmission of SARS-CoV-2 in English NHS Trusts: a computational modelling study. BMC Infect Dis. 2024;24(475). doi:10.1186/s12879-024-09330-z.

[3] Kazil J, Masad D, Crooks A. Utilizing Python for agent-based modeling: The Mesa framework. In: Thomson R, Bisgin H, Dancy C, Hyder A, Hussain M, eds. Social, Cultural, and Behavioral Modeling. Cham: Springer International Publishing; 2020:308-317.

[4] Bays, D., Whiteley, T., Williams, H., Finnie, T. and Gent, N., 2022. Mitigating isolation: further comparing the effect of LFD testing for early release from self-isolation for COVID-19 cases. MedRxiv, pp.2022-01.

[5] Quilty, B.J., Clifford, S., Hellewell, J., Russell, T.W., Kucharski, A.J., Flasche, S., Edmunds, W.J., Atkins, K.E., Foss, A.M., Waterlow, N.R. and Abbas, K., 2021. Quarantine and testing strategies in contact tracing for SARS-CoV-2: a modelling study. The Lancet Public Health, 6(3), pp.e175-e183

[6] Kissler, S.M., et al., 2021. Projecting the transmission dynamics of SARS-CoV-2 through the postpandemic period. The Lancet. Available at: <https://doi.org/10.1016/S0140-6736(20)30792-9> [Accessed 27 August 2024].

[7] Hall, V.J., et al., 2021. Effectiveness of BNT162b2 mRNA vaccine against infection and COVID-19 vaccine coverage in healthcare workers in England, multicentre prospective cohort study (the SIREN study). ScienceDirect. Available at: <https://doi.org/10.1016/j.jinf.2021.02.010> [Accessed 27 August 2024].

[8] Chitnis, N., et al., 2020. SEIR model for COVID-19 dynamics incorporating the environment and social distancing. BMC Research Notes. Available at: <https://doi.org/10.1186/s13104-020-05192-1> [Accessed 27 August 2024].

[9] UK Health Security Agency. Winter coronavirus (COVID-19) infection study: estimates of epidemiological characteristics, England and Scotland, 2023 to 2024: 21 December 2023. Available from: <https://www.gov.uk/government/statistics/winter-coronavirus-covid-19-infection-study-estimates-of-epidemiological-characteristics-england-and-scotland-2023-to-2024/winter-coronavirus-covid-19-infection-study-estimates-of-epidemiological-characteristics-21-december-2023>

[10] UK Health Security Agency. Winter coronavirus (COVID-19) infection study: estimates of epidemiological characteristics, England and Scotland, 2023 to 2024: 11 January 2024. Available from: <https://www.gov.uk/government/statistics/winter-coronavirus-covid-19-infection-study-estimates-of-epidemiological-characteristics-england-and-scotland-2023-to-2024/winter-coronavirus-covid-19-infection-study-estimates-of-epidemiological-characteristics-11-january-2024>

[11] UK Health Security Agency. Winter coronavirus (COVID-19) infection study: estimates of epidemiological characteristics, England and Scotland, 2023 to 2024: 15 February 2024. Available from: <https://www.gov.uk/government/statistics/winter-coronavirus-covid-19-infection-study-estimates-of-epidemiological-characteristics-england-and-scotland-2023-to-2024/winter-coronavirus-covid-19-infection-study-estimates-of-epidemiological-characteristics-15-february-2024>

[12] Andrews N, Tessier E, Stowe J, Gower C, Kirsebom F, Simmons R, et al. Vaccine effectiveness and duration of protection of Comirnaty, Vaxzevria and Spikevax against mild and severe COVID-19 in the UK. medRxiv. 2021. doi:10.1101/2021.09.15.21263583.

[13] UK Health Security Agency. Managing healthcare staff with symptoms of a respiratory infection or a positive COVID-19 test result [Internet]. GOV.UK; 2024 [cited 2024 Sep 26]. Available from: <https://www.gov.uk/government/publications/covid-19-managing-healthcare-staff-with-symptoms-of-a-respiratory-infection/managing-healthcare-staff-with-symptoms-of-a-respiratory-infection-or-a-positive-covid-19-test-result>

[14] Shang, W., Kang, L., Cao, G., Wang, Y., Gao, P., Liu, J. and Liu, M., 2022. Percentage of asymptomatic infections among SARS-CoV-2 omicron variant-positive individuals: a systematic review and meta-analysis. Vaccines, 10(7), p.1049.
